# Case of Penetrating Wound of the Thorax

**Published:** 1848-05

**Authors:** T. G. Meachem

**Affiliations:** East Bloomfield, Ontario Co., N. Y.


// BUFFALO MEDICAL JOURNAL

AND

MONTHLY REVIEW.

VOL. 3. MAY, 1848. NO. 12.

### ORIGINAL COMMUNICATIONS.

ART. 1.—*Case of Penetrating Wound of the Thorax.* By T. G. MEACHEM, M. D.

The subject of the following paper was a German, aged 21, of bilio-sanguine temperament, and strong vigorous constitution. In August last, while seated on a load of wheat descending a rough and uneven declivity, with a three-tined pitchfork loosely held in his hands, its points directed towards him, an unexpected jolt of the wagon suddenly threw him from his position to the ground, on to which, with an impulsive, but incautious effort to break the force of the fall, he attempted to rest the end of the fork, by which means his body was cast heavily on the opposing tines. So great was the momentum with which it met the instrument, that the stale was snapped short off, as though it had been a reed, about three inches from the socket; and so firmly was the remaining portion of the instrument set in the chest, that it was not without considerable difficulty that a fellow laborer succeeded in extracting it. As soon as possible the wounded man was conveyed home, from which, happily, he was not distant; and a messenger was immediately despatched for me. On arriving at the house, I found the patient, supported by two assistants, seated in a chair with his garments dyed in gore, and laboring under the following symptoms:—Complete aphonia; urgent dyspnœa and sense of suffocation; the head drooping, and the limbs relaxed; copious and cold perspiration; the countenance of a death-like pallor, and expression of agonizing anxiety; the pulsation of the radial artery almost extinct; immobility of the costal parietes, the abdominal muscles acting vicariously; and at every inspiration a gush of blood accompanied with bubbles of air, or followed by them, from the wounds in the chest. A single glance sufficed to show that he was in a state of extreme collapse; and satisfying myself by a hasty physical exploration of the thorax, that the prostration was not as much the result of internal hemorrhage as of the shock the system had received, I prescribed the usual diffusible stimuli, and rest in the horizontal posture. The latter part of the prescription, however, could not be tolerated for a moment, owing to the increase of dyspnœa and sense of suffocation it produced; and it was therefore countermanded. As soon as reaction had been partially effected, I proceeded to examine the nature and extent of the injury, and found three separated wounds on the right side of the chest; one situate above and a little to the right of the nipple, immediately over the third rib; the second directly below the nipple, between the fourth and fifth ribs; and the third between the fifth and sixth ribs, in proximity to the sternum. The first puncture, judging from its position—*over the rib*—as well as from the absence of hemorrhage and the non-escapement of air from it, evidently did not extend into the cavity of the thorax, and was consequently a mere flesh wound. The second wound, however, from the presence of those symptoms absent in the first instance, must not only have pierced the thoracic cavity, but must have involved the lung itself; though, from the fact that the tine which caused it had been broken at its point prior to the accident, probably not to any great extent. The third wound, on the contrary, was of a highly serious character; for, reasoning from all the circumstances attending it, it must have penetrated the chest to the depth at least of two and a half or three inches. They were all in an oblique direction downward, the fork having come into collision with the chest with its convexity upward. Seeing the grave nature of the case, and wishing to give my patient the benefit of united medical experience, I sent for my friend, Dr. Webster, of East Bloomfield, in consultation. In the mean time, reaction being pretty well established, I drew 12 oz. of blood from the arm, which produced a decided melioration of the suffering and distress. On conferring with the Dr., it was resolved to close the apertures, and apply a firm bandage to the thorax to prevent motion of the ribs, the practice recommended by Hennen, Druit, and others; and having, in addition, enjoined on the patient through his friends the necessity of perfect quietude and strict adherence to the antiphlogistic regimen, we left, with a promise on my part to see him again in a few hours.

11 *o'clock*, *P. M.* Reaction again taken place, the skin having become hot, the respiration labored and quick, the pulse full, hard and rapid, &c., &c. Slight dulness on percussion over the right dorsal, lateral, and mammary regions, together with a diminution of the respiratory murmur in the same regions, phenomena due, doubtless, to the effusion of blood into the pleural sac; but as the patient could not be moved without experiencing great pain, the fact whether or not they shifted their site according to the position he might assume, could not be ascertain. ℞, V. S. 24℥. I staid with him during the night.

*Thursday* 8, *A. M.* Has passed an uncomfortable night, having been unable to sleep but a few minutes at a time. Febrile action again on the increase, though it has not attained the point at which it was last combatted by bleedings. A white coat forming on the tongue. Some degree of cough present, unattended by expectoration. No change in the physical signs. ℞. V. S. 16℥; after which, sulph. mag. 1℥.

4, *P. M.* No operation of the cathartic. Pulse changed but little since morning; by no means bad relatively. Inability to lie down in the recumbent or lateral positions. Pain in the chest on inspiration. Has urinated freely, the urine being somewhat heightened in color. Considerable thirst, though not much anorexia. Tongue the same; and no mentionable alteration in the physical signs. ℞. sulph. mag. 1℥. to be repeated in six hours, if necessary.

*Friday*, 10. *A. M.* General symptoms the same as yesterday. No movement of the bowels. The bandage having become loose, I removed it for re-adjustment, taking occasion to examine more closely than I could otherwise do, the physical condition of the chest. The right side was far less mobile than the left. On applying my ear to the mammary region of the right side, an unusually loud, clear, and, apparently, *near* crepitation was audible on inspiration, for which, at first, I could hardly account; but palpation of the integuments soon solved the mystery by revealing a slight emphysema in the part, so that the air effused into the cellular tissue, had, at every upward movement of the thoracic parietes, been compressed against my ear, necessarily producing an *abnormal* crepitation, if I may so term it. Not having seen this source of error alluded to in any treatise on the physical diagnosis of diseases of the chest, (although the abstract fact that emphysema will cause a crepitation on pressure of the part affected, and is, indeed, to be recognized in that manner, is mentioned by all), I was not, I must confess, prepared to meet with it, especially as the emphysema was *so* slight as to occasion no visible tumor or distension. The lesson of caution, however, which all such instances should teach the surgeon, is one certainly worth remembering; for, without uncharitableness, I may venture to say that a hasty, superficial, or ignorant examiner might easily be led astray by a phenomenon much simpler than this, which, by such a one, would probably have been located in the lung instead of the teguments of chest: How widely different the pathology of the two cases, and how *toto coelo* dissimilar their resulting treatment! As might have been anticipated, this emphysemal crepitation interfered very much with the recognition of the physical sounds of the lung: in fact, over the small space which it occupied, it almost entirely masked them, rendering the information they might otherwise have afforded, nugatory. To the right of the mammary region, where the emphysema did not encroach, the minute crepitation of commencing pneumonia, or pneumonic crackling, was quite perceptible, gradually diminishing in intensity as the ear traveled from the mammary into the lateral region. With the exception of the diminished respiration already referred to in the lower portion of the dorsal region, the respiration in that part was normal. Percussion over this region gave the dulness previously spoken of; over the lateral region a dulness steadily increasing as you approached the sternum, proportionably with the decrease of the respiratory murmur and the augmentation of pneumonic crackling; while on the mammary region its employment was wholly prohibited, on account of the tenderness and pain present from the inflammatory action going on in it. The remaining regions of the right side of the thorax yielded their usual healthy signs, with the exception of a diminished force of the vesicular murmur. Thoughout the left side of the chest puerile respiration was well marked. As the degree of pneumonia existing was no greater than might naturally have been expected, and, perhaps, desired for the reparation of injured parts, I thought it best not to prescribe for it directly, but, resolving to watch it, I ordered the following medicine: infusion of senna with salts (the senna tea of U. S. Dispen.) 2℥ every three hours, until it operated.

5 *P. M.* No particular change ; and no action of the bowels. Complains of hunger and faintness. Some boborygmus. ℞: infusion of senna with salts once in two hours, with an occasional enema; and may be allowed at intervals a small quantity of thin water porridge.

*Saturday*, 11, *A. M.* Cathartic operated well. Symptoms of pneumonia stronger and more decided than yesterday, which the physical signs corroborate. ℞: sol. gum acac., nit. pot., and tart. antim., once in three hours.

*Sunday*, 12*th*. Pneumonia unabated. In the course of the night the bloody pathognomonic sputa began to be expectorated. More pain on inspiration than heretofore. Pulse about 80, full and quick, but soft. Continue solution every two hours.

*Monday* 13*th*, *A. M.* Much improved in every respect. From this time, under the moderate use of ipecac, calomel, and Dover's powder, with an occasional laxative, he steadily convalesced, though it was not for several weeks after he was able to follow his ordinary avocation, that the injured lung appeared to have regained its normal state.

The after treatment of this case strongly suggested to my mind the following query: Why would not *iodine*, in some of its numerous forms, be a valuable remedy in removing liquids effused into the pleural cavity? Reasoning analogically, it most undoubtedly would; for in producing absorption of effused fluids in other serous sacs, as in the instances of ovarian dropsy, ascites, and hydrocephalus, its virtues are far from hypothetical. If I mistake not, it was the reputed sanatory influence of nit. arg. in solution over certain inflammations of the mucous membrane of the mouth, fauces, &c., that led to its trial in dysentery, diarrhœa, and laryngitis. Why may not an experiment with *iodine*, based on similar principles, establish *its* efficacy in hydrothorax or even hemathorax? Has the matter ever been satisfactorily tested? If so, our standard authors are culpably silent on the point.*

East Bloomfield, Ontario Co., N. Y. March, 1848.

* Iodine, in chronic pleuritis with effusion, is strongly recommended by Dr Stokes, of Dublin. Dr. S. gives this article preference over any other, as a sorbefacient in that disease. He recommends it to be administered internally, and, at the same time, applied externally over the chest.—Editor.

